# “I Wouldn’t Even Know What to Ask for”: Patients’ and Caregivers’ Experiences of Psychological Support for Huntington’s Disease in Italy

**DOI:** 10.3390/neurosci5020007

**Published:** 2024-03-28

**Authors:** Nicolò Zarotti, Barbara D’Alessio, Marta Scocchia, Melissa Casella, Ferdinando Squitieri

**Affiliations:** 1 Division of Health Research, Faculty of Health and Medicine, Lancaster University, Lancaster LA14AT, UK; 2 Department of Clinical Neuropsychology, Manchester Centre for Clinical Neurosciences, Salford M68HD, UK; 3 Centre for Neurological Rare Diseases (CMNR) of LIRH Foundation, 00161 Rome, Italy; barbara.dalessio@lirh.it (B.D.); marta.scocchia@lirh.it (M.S.); melissa.casella@lirh.it (M.C.); ferdinando.squitieri@lirh.it (F.S.); 4 Huntington and Rare Diseases Unit, IRCCS Casa Sollievo della Sofferenza Research Hospital, San Giovanni Rotondo, 71013 Foggia, Italy

**Keywords:** Huntington’s disease, clinical psychology, psychological support, psychotherapy, Italy

## Abstract

People with Huntington’s disease (HD) often experience psychological difficulties linked with disease progression and the adjustment to living with a chronic condition, which are also frequently shared by their informal caregivers (e.g., partners). Although limited, the current literature on psychological care for people with HD shows that interventions have the potential to drive improvements in mental health and quality of life. However, the experience of accessing and receiving psychological support for HD remains unclear across several countries. This study adopted a qualitative design to explore the experiences of psychological support for HD from the perspectives of patients and caregivers living in Italy. Semi-structured interviews were carried out with 14 participants—7 patients with early-manifest HD and 7 partners acting as their caregivers. The resulting data were analysed through thematic analysis. Four overarching themes were identified: (1) the availability of psychological support for HD, (2) barriers to accessing psychological support, (3) enablers to accessing psychological support, and (4) the future development of public psychological provision for HD. In Italy, patients and caregivers perceive public psychological support for HD as unavailable or inadequate, and private therapy is often seen as unaffordable. Barriers such as distrust in public healthcare and preconceptions about therapy may limit access, while advice from HD organisations and seeking therapy for other reasons may act as enablers. A strong emphasis is put on the need for accessible public psychological support throughout all the stages of the condition.

## 1. Introduction

Huntington’s disease (HD) is a hereditary condition characterised by a genetic mutation that leads to extensive damage to the basal ganglia [1]. This results in severe motor impairments—including involuntary movements, ultimately leading to complete loss of ambulation and speech [2,3]—as well as cognitive deficits, such as problems with executive functioning and social cognition [4,5,6]. HD follows an autosomal-dominant inheritance pattern, with each afflicted individual having a 50% chance of passing on the condition to their offspring. It is considered a rare disease, with a prevalence of approximately 3.92 per 100,000 worldwide [7].

A genetic test, available since 1993, allows individuals with a family history to detect the mutated gene before motor symptoms manifest themselves [8]. However, this cannot predict the precise time of onset, which usually occurs between ages 35 and 45—although juvenile onset (i.e., before age 20) is also possible [9]. As no cure has been discovered to date, life expectancy post-diagnosis spans between 15 and 20 years on average [10,11]. The disease trajectory has been traditionally divided into five stages [12], with Stage I (or ‘early stage’) being characterised by mild motor and cognitive impairments, and Stage V (or ‘late stage’) requiring full-time care due to severe motor impairments and dementia.

The manifestations of Huntington’s disease (HD) are often associated with a large number of psychological difficulties [13]. Among these, the most frequent are depression, anxiety, irritability, aggressiveness, compulsions, and apathy [14], Around 13% of people with HD (pwHD) may also display obsessive–compulsive behaviours [15], while psychotic issues such as delusions and hallucinations tend to be rarer [2,16]. Such difficulties are also accompanied by an increased risk of suicidal tendencies in both premanifest and manifest individuals [17].

With regards to quality of life, depressive symptoms and cognitive impairments are often reported as being more determining by pwHD [18], even more so than physical symptoms [19,20]. Furthermore, since the result of the predictive test for Huntington’s can only confirm whether an individual carries the expanded HTT gene but cannot specify when the onset of symptoms will occur, the psychological impact of genetic testing represents another significant challenge [21], and a positive test result can, in some cases, be associated with suicidal ideation [22]. Being a disease of families, HD can also lead to significant psychological issues linked to growing up with affected relatives (e.g., attachment problems [23]), as well communication difficulties around discussing the family history of the disease, especially with young children [24].

To address the issues above, a novel drive towards developing forms of psychological support for pwHD has recently started gaining momentum, particularly with the aim of introducing a psychologically informed clinical framework to the understanding of distress in this population [25,26]. As a direct consequence, a number of psychological interventions have been also begun to be adapted and trialled for HD, showing positive preliminary results [27,28,29,30]. However, the overall evidence on the use of such interventions in this population remains extremely limited. More specifically, a recent literature review [13], echoed by guidelines published in the UK by the British Psychological Society [31], identified an alarming lack of data on the subject and a consequent scarcity of dedicated psychological services. 

These shortcomings appear to be especially relevant in Italy, where no investigation of psychological interventions for HD has been carried out so far [13] and where the current availability of mental health services for HD, and their presence in communities, are far from clear. Similarly, the subjective experience of Italian patients and caregivers receiving some form of psychological support remains unknown. Thus, the main objective of this study was to explore the subjective experience of HD patients and caregivers with regards to accessing psychological support for HD in Italy. More specifically, this study aimed to answer the following research question: *What is the subjective experience of psychological support for individuals with Huntington’s disease and their caregivers in Italy?*

## 2. Materials and Methods

### 2.1. Methodological Approach

This project adopted a qualitative methodology based on a semi-structured interview approach [32,33]. 

### 2.2. Sampling and Recruitment

Convenience sampling was used, whereby Italian HD patients under the care of the Italian Huntington’s Research League (LIRH) Foundation—an Italian not-for-profit organisation founded by patients, clinicians, and researchers with the aim of improving the quality of life of people with HD—were invited to participate, along with their partners acting as informal caregivers. Due to the significant cognitive and communication difficulties that are likely to arise at later stages of the condition [34], only patients in the early-to-intermediate manifest stage (e.g., Stage I–II) were invited. To facilitate engagement, participants were given the option of choosing between individual or dyadic interviews.

### 2.3. Participants

Eligible participants were approached in person during routine outpatient clinics held in 2022 at the Neurological Rare Disease Centre of the LIRH Foundation in Rome, Italy. During these appointments, potential patients and their accompanying caregivers were provided detailed information about the purpose and methods of the study by their neurologist (FS) or clinical psychologist (MS, MC). The opportunity to ask for any clarifications was also offered at this stage. 

Of 20 participants approached initially (10 patients and 10 caregivers), 14 eventually accepted taking part in the study and agreed to be interviewed. These included seven patients with early-manifest HD and seven caregivers. Two participants were living in Northern Italy, eight in Central Italy, and six in Southern Italy. Table 1 illustrates the basic demographic characteristics of all participants. 

### 2.4. Procedure

The interviews were conducted online between April and August 2022 via the Zoom^®^ platform (v. 5.11). These were all carried out by NZ, a doctoral-level clinical psychologist with more than 10 years of experience in qualitative research and no previous clinical involvement with the participating patients and caregivers. Based on the participants’ preference, eight individual interviews and three dyadic interviews were conducted. Each interview lasted 48 min on average (range: 39 to 59 min) and was carried out according to a 4-point schedule developed by the authors through iterative discussions and consultations (see Table 2 for a summary). 

### 2.5. Data Analysis

All interviews were audio-recorded, transcribed verbatim, and imported into NVivo^®^ qualitative software (v. 14), where they were then analysed thematically [35]. Thematic analysis (TA) [36] was deemed the most appropriate method, since it allows for both a deductive and inductive approach befitting the exploratory nature of the present study [36,37].

Based on the principles for TA outlined by Braun and Clarke [35,36,37], the analysis process started with multiple readings of the transcripts in order to allow for a familiarisation with the data and the annotation of initial ideas. Following this, a list of initial codes was identified and grouped into preliminary themes. The latter were then reviewed to ensure consistency with the codes (Level 1) and the full dataset (Level 2). Clear names and definitions were subsequently specified for each theme, and a thematic map was generated. Finally, all the findings were summarised in a report which included a selection of relevant verbatim quotes from the interviews. The formal analysis was initially carried out by NZ and then discussed carefully with all the other authors to ensure an adequate level of rigour and trustworthiness in the TA (e.g., transparency, credibility, and reliability) [38]. Throughout the analysis, the authors held a critical realist epistemological stance, recognising people’s experiences as equally real and meaningful as physical and behavioural phenomena [39]. 

### 2.6. Ethical Considerations

Formal ethics approval for the present study was granted by the Institutional Review Board of the LIRH Foundation (Protocol no 5.300921 of 30 September 2021). Written informed consent was obtained from all participants. 

## 3. Results

### 3.1. Overview of Identified Themes and Codes

**Figure 1:**
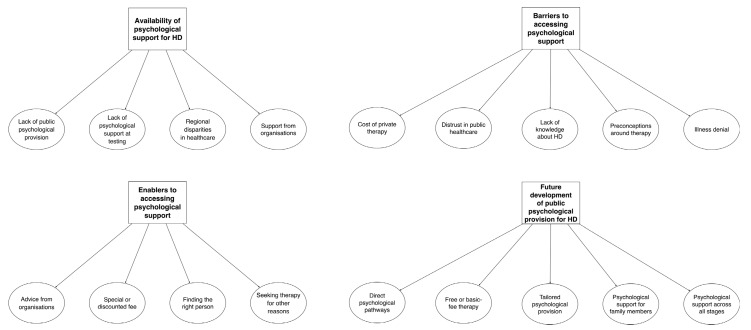
Final thematic map.

Initially, a preliminary list of 36 codes was generated. Following the abovementioned review of Level 1 and Level 2, 18 final codes were eventually identified and organised into 4 overarching themes. Table 3 provides a summary of the main themes and respective codes, while Figure 1 illustrates the final thematic map. The findings of the study are outlined below, along with relevant quotes from the interviews. The names of specific codes are highlighted in italics within the text. 

### 3.2. Theme 1—‘Unfortunately, That’s Our Country for You’: Availability of Psychological Support in HD

The first major theme to emerge from the interviews revolved around the participants’ views on the availability of psychological support for HD in Italy. More specifically, many felt that such support was unavailable or inadequate due to a *lack of public psychological provision* in general:

*In my opinion, the big problem is public psychological support in general, not just for HD. Because I did not receive any psychological support even after I suffered a heart attack, after spending a week in the ICU. […] Public psychological provision is extremely scarce.*

—Caregiver 7

*It’s almost laughable, non-existent. At least in my case, sadly.*

—Patient 7

*There are no services around here. Nothing.*

—Caregiver 3

For some participants, the idea that psychological provision was lacking appeared to have originated from negative experiences during the HD diagnostic process, and specifically from receiving *no psychological support at testing*—an occasion where they felt left alone to deal with life-changing news:

*I had my genetic test in 2010. It was me, my sister, and my newlywed wife. They gave us the results and only said ‘as you know, the disease can be inherited with a 50% chance’. My sister was fine, I had the gene. […] And that was it. I did not receive any psychological support; they just gave me the unvarnished truth. It made me feel distraught.*

—Patient 1

*I wasn’t expecting them to play it down, but they also gave me the results making me feel as if I was just about to die, as if it was the most horrible news in the world. It felt devastating and overwhelming.*

—Patient 5

*The way they delivered the news really was really traumatic. There was no psychological support, they simply told my husband: ‘your sister is fine, you are sick’.*

—Caregiver 1

*They told me: ‘Just put this envelope [the results] in a drawer and get on with your life’. But that’s not how it goes, obviously. […] It’s terrifying. Absolutely terrifying.*

—Patient 2

In addition, since the Italian healthcare system is highly decentralised and administered on a devolved regional level [40], the availability of psychological support was believed to vary significantly across the country due to *regional disparities in healthcare*. This appeared to induce a number of participants to feel that the lack of provision was, in fact, more of a local issue in their region rather than a nationwide problem:

*Unfortunately, Italy is split into two, you know. In the north certain things work better, despite all the country’s problems, and the centre still manages to make do. But if you go south, things get pretty difficult, I believe.*

—Caregiver 7

*I don’t think public psychological services can help you, because we don’t have enough psychologists. […] Our local orthopaedics department is shutting down because they are out of surgeons. We have no orthopaedic doctors, let alone psychologists.*

—Patient 3

*I believe psychological services for people who have difficulties are more present in regions where public healthcare functions better. […] As long as we have this patchworked Health Service, with different rules and wait times across regions, it’ll always be hard to reach some equality. Unfortunately, that’s our country for you.*

—Caregiver 5

On the other hand, while not consisting of any formal psychological intervention, the *support from organisations* dedicated to HD appeared to represent at least an informal source of psychological support for some patients and caregivers:

*If I need anything, I know I have my HD organisation. They call me often, roughly once a month, to check in on me. They always tell me to let them know straight away if there’s something going on, to call them. So, I feel looked after.*

—Patient 3

*They told me that they were there for me, if I needed anything. That I could call them anytime.*

—Patient 6

*As far as I know, the psychological support for HD is left to non-profit organisations which take care of patients in the community.*

—Caregiver 5

Despite the informal role played by HD organisations, however, the combination of public provision issues mentioned above appeared to discourage some participants from seeking public support in the first place, even when they felt it would be helpful:

*I did not ask for [public] support. First of all, because I don’t believe we have any services around here. I wouldn’t even know what to ask for. I don’t think there are any services that could help.*

—Patient 3

### 3.3. Theme 2—‘I’m Afraid of Seeing Someone Who Doesn’t Know HD’: Barriers to Accessing Psychological Support

When some form of psychological support was available to participants, a number of factors appeared to act as barriers to their ability or motivation to access it. For instance, while seeing a private psychologist was considered an option by some patients and caregivers, the significant *cost of private therapy* represented a limitation for many:

*I went to see a psychologist a couple of times. But then I stopped because my partner was changing his job and we could not afford it.*

—Patient 6

*To be honest, when it comes to [therapy] costs… well, 70 euros per week […] it’s hard. Even though it’s helpful. Very helpful.*

—Caregiver 7

*I used to see a private psychologist for a few years. [Then] I lost my job, so I did not have the money to continue.*

—Patient 7

To some, private therapy felt even less affordable, since other family members were already seeing a private psychologist, making it stressful to consider adding further weight on the family budget:

*Personally, I could use some form of psychological help, but I can’t afford it. One of my daughters is already seeing a private psychologist, so I cannot afford two private therapies.*

—Patient 5

*We get along just fine, but we can’t afford to spend 1000 euros a month to pay for therapy for the whole family.*

—Caregiver 1

In cases where public psychological support was available, however, numerous participants still reported feeling reluctant to seek help. This appeared to be due to a *distrust in public healthcare* linked to multiple systemic issues, such as long waitlists and lack of organisation:

*Public healthcare is a disaster. It doesn’t work well. When we go to the hospital, for example, it’s a mess.*

—Caregiver 6

*Unfortunately, waitlists for public psychological support are much longer.*

—Patient 7

*The whole healthcare system needs to be reformed because it’s getting worse across the board.*

—Caregiver 5

At times, this feeling of distrust also appeared to be fuelled by advice received from other people based on incorrect assumptions, such as that psychologists would prescribe pharmacological treatments:

*We were advised to avoid psychologists in the public healthcare system, because they just fill you up with medications.*

—Patient 6

Another factor playing a pivotal role in shaping the participants’ motivation to access public psychological support was represented by the general *lack of knowledge about HD* which both patients and caregivers felt characterised clinicians in the Italian healthcare system. More specifically, many reported having to explain the disease to their psychologist or physician, which led to increased feelings of frustration and a decreased motivation to engage with services:

*I saw a psychologist; she barely knew what HD was. She said to me: ‘Oh, I’ve heard about it once”. What do you mean you’ve heard about it once? That’s when I left.*

—Patient 7

*If you go see a psychologist here, they won’t know what HD is. […] I’ve recently met my new GP, I told her my diagnosis, and she didn’t know what it was. After this she did catch up with the literature. But my GP didn’t know what disease it was!*

—Patient 3

*I’m afraid of seeing someone who doesn’t know HD or knows little about it. ALS and Alzheimer’s are known, so you have centres, psychological services dedicated to them.*

—Patient 6

In addition, several *preconceptions around therapy*, irrespective of its public or private nature, also emerged as further barriers to accessing help. For example, some of the participants felt that psychological help was only targeted at individuals with severe mental health difficulties, especially when accessed within the national healthcare system:

*Well, I believe that psychological support only really exists for people who have severe issues.*

—Patient 5

*The national healthcare system only offers sporadic help, and you need to have some severe problem. I am not severe enough, I’m too stable! [laughs]*

—Caregiver 1

Some also appeared to see the experience of therapy as not fitting specific mindsets or gender roles, particularly due to the stigma attached both to therapy and HD in certain contexts or environments:

*My dad would have never accepted any psychological help. Because he felt he ‘was a man’, you know.*

—Patient 7

*Up until a couple of years ago, if you told my husband about seeing a psychologist, he would tell you it was money down the drain. [...] It’s just this small-town mindset, you know—if you see a psychologist, it means you’re a fool.*

—Caregiver 1

Others instead highlighted how they did not feel themselves to be the best candidates to receive psychological support due to personality traits, such as having a tendency to be introverted or reserved:

*I am not a person to work on his own subconscious, I don’t dig deep inside. So I don’t feel like having any psychological support for now.*

—Patient 1

*My character is quite reserved, I am a bit withdrawn. So, I don’t like discussing my business, my feelings, or my problems with other people. I find my support within my family, with my sisters or my mother.*

—Caregiver 3

This also showed the potential to be further exacerbated by previous negative experiences of therapy, which in turn would act as a barrier to accessing support after receiving the HD diagnosis:

*I’ve had, let’s say, a bad experience with psychological support. I was forced to engage with it in 2019, due to some difficulties with my son. [...] My wife and I were sent to see a family psychologist once a week, and this never really sat right with me. [...] I felt helped a bit, but then this woman would dig up my past every time, and I always felt I had to open up too much. [...] And in 2021, when I received my diagnosis, I was advised to see a psychologist. But I’ll be honest, I did not do it. I did not follow that advice, coming from my previous experience.*

—Patient 1

Finally, a number of caregivers also highlighted how their partners’ willingness to access psychological support was sometimes affected by *illness denial*, whereby patients rejected the idea of being ill and thus also any need for help with the psychological adjustment to the condition:

*Receiving some psychological support is always good, so I would accept it if it was offered to me. But my wife? I don’t know, she is a bit peculiar about this. […] Because she rejects the disease at times. She doesn’t want to feel ill, let’s say.*

—Caregiver 5

*My husband denies it, but we were only married three months when we found out [about HD]. And I married a cheerful guy who turned into another person overnight. Always serious and withdrawn. […] All these years, he has kept telling himself this fairy tale that he has not changed. […] A defence mechanism, isn’t it? […] And I keep telling him: ‘You need to get some help!’*

—Caregiver 1

### 3.4. Theme 3—‘I Would Speak with My HD Organisation First’: Enablers to Accessing Psychological Support

Despite the barriers they faced with regards to accessing psychological support, many participants also highlighted several enablers that appeared to facilitate this process, which emerged as the third overarching theme. For instance, a number of patients and caregivers saw in the *advice from organisations* a valuable form of encouragement towards connecting with a psychologist. Sometimes, this took the form of word-of-mouth recommendations of a clinician who, albeit privately, would be well-equipped to work with a patient with HD:

*The HD organisation’s staff gave me the contact details of a fantastic psychologist.*

—Patient 7

*The organisation’s staff recommended this psychologist, in case I wanted to receive some support, because they knew her. So, I went to see her.*

—Patient 6

For those who struggled to afford private therapy, this process also appeared to be further facilitated by receiving *special or discounted fees*:

*The psychologist was private, […] but we agreed a discounted fee.*

—Patient 6

*The psychologist gave me a special treatment. The regular fee was 70 euros per session, I only paid 45. It was a fantastic deal.*

—Patient 7

The participants who had yet to engage with psychological support, instead appeared to keep in mind the potential for asking their organisations for advice in case they decided to undergo therapy in the future:

*If I need it, I’ll certainly seek some support. […] But I would speak with my HD organisation first, about seeing a psychologist.*

—Patient 2

*If my HD organisation has the contact details of someone in my area, someone they know, who knows HD… then this person would already know what I’m talking about.*

—Caregiver 4

Another important factor in shaping the participants’ motivation to access psychological support revolved around *finding the right person*, which many considered essential to be able to engage meaningfully with psychological work, even if it meant having to compromise:

*First of all, I need to be able to choose the person to do therapy with. They can’t be someone random, because rapport is essential in therapy, the empathy between the patient and the clinician.*

—Patient 5

*Someone who knows HD, is welcoming, and understands our difficulties. […] I’ll go where I need to. If it has to be a private one, then I’ll see a private one.*

—Patient 4

*I was really lucky to find the perfect psychologist for me from day one. Love at first sight, you know. And that helped me massively.*

—Caregiver 1

*You know what matters the most to me? Trust. If I trust a person, I’ll be happy to see them even online.*

—Patient 2

While most participants discussed engagement with psychological support in relation to HD, some identified a further enabler in *seeking therapy for other reasons*. For instance, a number of participants mentioned having a positive attitude towards therapy thanks to having sought help due to other life events or difficulties:

*I saw a psychologist previously, but for different reasons. […] a friend of mine died by suicide, and I felt like giving everything up. So, I told myself: ‘I need some help’. And I started seeing a psychologist every week. […] It was the best choice I could ever make.*

—Caregiver 1

*I went to see a psychologist for other problems, some problems I had with my daughter. […] It helped me increase my self-esteem.*

—Patient 2

In one case, this also motivated a patient to reconnect with her previous psychologist after she received her HD diagnosis, even if just for one session:

*When I got the news [diagnosis], I reconnected with the psychologist I saw for a long time when I was at university. I went to see him only once. […] One session was enough for me, to ‘reset my mind’.*

—Patient 5

### 3.5. Theme 4—‘We Should Just Be Able to Call’: Future Development of Public Psychological Provision in HD

The final theme to emerge from the interviews concerned patients’ and caregivers’ views on how to develop public psychological support for HD in the future. In this regard, a first change which many participants identified as essential was the development of *direct psychological pathways* that would allow people with HD and their caregivers to access psychological help on their own, without having to ask other professionals to refer them. For instance, some thought this could take the form of a hotline:

*A toll-free number to support patients and caregivers at different life stages. When they are having acute difficulties, because these can happen, but also during moments of apparent calm.*

—Caregiver 5

*We should just be able to call… ring a person who would be able to see how things are going, what patients need.*

—Caregiver 6

In addition, the development of easy-access specialised walk-in centres or services was also seen as a potential way forward, albeit not without compromises:

*They should create ad-hoc centres. […] Listening or counselling centres. There may not be enough resources for ones dedicated to Huntington’s disease, because it’s a rare condition, but they could create some regional centres for rare neurodegenerative diseases where patients can access psychological support.*

—Caregiver 5

*It needs to be easy to access. Being public, it needs to be a service where if you ask for a session today, they don’t tell you: ‘Come the day before Christmas’. Because that’s how public services work right now.*

—Caregiver 2

As many participants highlighted, such services would also need to offer *free or basic fee therapy,* to allow people who cannot afford private support to access them:

*It should be free! [laughs]*

—Patient 4

*To have a support… that is public and doesn’t break the bank. That’d be helpful.*

—Patient 6

*It should be free, or with a fee based on income. […] So that those who can’t afford it don’t need to pay.*

—Caregiver 5

In terms of content and modalities, participants expressed a desire for a *tailored provision* that would account for the wide range of needs that different individuals may have. For instance, some highlighted how home visits or online sessions may be helpful for those with mobility issues, especially to avoid relying on their caregivers:

*For someone like my dad, who has trouble getting out of the house, it should be possible to do it online or to have a home visit. It should be tailored around a person’s needs.*

—Patient 5

*If it’s public, it also needs to be accessible from home without having to travel here and there. That can also be a problem, you know. [...] My wife currently goes on her own [to therapy], but if we both need to start traveling there then it can become a problem.*

—Caregiver 2

Others also stressed how public psychological provision would have to be tailored more around needs specific to HD and be offered by clinicians who are familiar with and have previous experience of the disease:

*We need psychological support from someone who knows the condition, who knows other cases. Someone experienced.*

—Caregiver 3

*It would take someone who knows the disease and, most importantly, has had some experience dealing with a patient with Huntington’s.*

—Patient 3

With regards to this, one of the HD-specific needs which emerged most consistently across participants was the opportunity to receive *psychological support for family members.* For example, many felt they could benefit from psychological help when discussing HD with their children:

*When the time comes to tell the children [about HD]. Then, yes, a psychologist would be helpful, maybe even a developmental psychologist.*

—Caregiver 4

*Maybe a psychologist would be better than me at dealing with these things, they are able to connect with the kids, use the right words.*

—Patient 4

This appeared especially important in the longer term, as having HD running in the family also seemed to extend some participants’ perceived parental responsibilities beyond the early-adult stage:

*I don’t know if due to the disease or what else, but we are living through a time of enormous change. […] The girls are growing up and I thought that my role as a parent, you know, I didn’t think it would end exactly when they turn 18, but I thought it would get at least a bit lighter. Instead, I feel a great pressure to continue supporting my daughters.*

—Patient 5

Similarly, the availability of psychological support for partners acting as caregivers of people with HD was seen as essential, especially when dealing with challenging behaviours such as irritability and aggressiveness:

*We need clinicians who are qualified to work psychologically with patients as well as partners and relatives […] because at times they may struggle to understand that issues such as irritability and aggressiveness are due to the disease.*

—Patient 3

*They [psychologists] would be needed to help caregivers understand that life isn’t over. I see my father-in-law and my heart bleeds. […] There should be some support for family members too.*

—Patient 5

Finally, the specific timing of psychological support in HD was also considered crucial by numerous participants. More specifically, many identified a need for *psychological provision across all stages* of the condition—starting with genetic testing and the clinical diagnosis stage:

*It needs to be available before taking the test. When you go and do the test you must be sure, psychologically, that you want to do it. Because otherwise you risk being hit by a brick.*

—Patient 1

*It’d be certainly very important in the beginning, when it’s diagnosed. It’s life-changing, so a psychologist would be needed for sure.*

—Caregiver 4

A few others also highlighted how receiving some form of psychological support would be helpful for difficulties that may be experienced at the moderate and late stages as well, such as suicidal ideation and end-of-life care:

*Psychological support would also be needed halfway through… after someone gets to know, because then you get the thought… personally, I’ve never had the idea of taking my own life, because I never thought about it. But there are people who may.*

—Patient 6

*Perhaps it would be good to get some help during the final stage, when maybe you start realising you are about to go and you need support.*

—Patient 4

Ultimately, the timing of provision appeared to be seen as linked to its required tailored nature, i.e., allowing for people with HD and their caregivers to ask for help at different stages, as their personal circumstances and specific needs evolve across the disease trajectory:

*I would ask for psychological help according to my needs, and I believe needs increase over time. So, maybe now I need it less than I will in one, five, or ten years.*

—Caregiver 7

*It has to be a type of support that evolves along with the disease, because each different situation needs a different type of intervention.*

—Caregiver 2

## 4. Discussion

### 4.1. Summary of Main Findings

This study explored the experiences of psychological support for Huntington’s disease (HD) from the perspectives of patients and caregivers living in Italy. To our knowledge, this is the first study to date to investigate this topic. A series of qualitative semi-structured interviews were carried out with seven patients with early-manifest HD and seven partners acting as their caregivers. The resulting data were analysed through thematic analysis, which identified four overarching themes: (1) the availability of psychological support for HD, (2) barriers to accessing psychological support, (3) enablers to accessing psychological support, and (4) the future development of public psychological provision for HD. To our knowledge, this is the first investigation to date to address this under-researched topic in Italy.

The first theme concerned how people with HD and their caregivers experienced the availability of psychological support for the condition. This was seen to be unavailable or inadequate by most participants, particularly due problems such as a general lack of public psychological provision, no psychological help at the testing stage, and regional disparities in how healthcare is administered. However, participants also reported that HD organisations appeared to provide informal psychological support when none was available within the national healthcare system. Problems around the limited public provision of psychological support in the Italian healthcare system have been reported previously [41]. In addition, some of these findings appear to be in line with studies carried out in other countries such as the United Kingdom, which reported issues with patchy and unequal provision for HD [25,26,42], often affected by ‘postcode lotteries’ dictating service availability [43].

The second theme revolved around barriers to accessing psychological help for HD, which included the cost of private therapy, a generalised distrust in public healthcare, lack of knowledge about HD, illness denial, and preconceptions around therapy. Again, part of these findings appears to be consistent with the current literature, not only in HD but also other populations as well. For instance, a general lack of trust towards healthcare (particularly in some regions) has been observed before among Italians [44], and the cost of therapy, preconceptions or concerns around being in therapy, and illness denial have all been reported previously as barriers to accessing psychological support [45,46,47]. Similarly, the issue of healthcare providers lacking knowledge of HD and its negative impact on affected individuals and their caregivers has been documented extensively [48,49,50,51].

In contrast to the barriers mentioned above, participants also reported several enablers of access to psychological support. These formed the third theme, and included receiving advice from HD organisations, finding the right clinician for therapy, seeking therapy for reasons other than HD, and receiving special or discounted fees. With regard to these, the stakeholder role played by HD organisations in our study appeared consistent with previous evidence showing that patient associations can be instrumental in facilitating access to healthcare treatments in general [52,53]. Moreover, special arrangements or support to face the costs of private therapy, and the rapid development of a positive rapport with a psychologist, have both been reported previously as enablers [47].

Finally, the fourth theme to emerge explored the views of patients and their caregivers around the future of Italian public psychological provision for HD. Overall, most participants saw the development of direct psychological pathways—providing free (or basic fee) tailored psychological support to both patients and their family members across all stages of the disease—as the right way forward. All these elements appeared to be largely in line with evidence from other neurodegenerative conditions [54], as well as with some of the suggestions outlined by HD guidelines and manifestos published recently in the UK, which also highlighted the need for exploring national psychological provision for HD in other countries [25,26,31].

### 4.2. Clinical Implications

Several clinical implications may be drawn from the results of the present study. First, a renewed emphasis should be placed on increasing clinicians’ knowledge and education around HD in Italy. This should be done in liaison with national and international HD organisations, which should also be aware of the pivotal role they may play in terms of facilitating patients’ and caregiver’ access to and trust in psychological support at large. Secondly, clinicians should be more aware of the different psychological requirements experienced by patients at different stages of the condition, and they should aim at providing constant and reliable support across the whole disease trajectory. A drive towards tailoring psychological provision around not only HD-specific needs but also personal circumstances should be emphasised as well—for instance, by offering online therapy or home visits to those with mobility issues. Finally, the adoption of psychologically informed systemic approaches, which recognise the need to provide psychological support to family members and promote therapeutic work involving the entire family system, should be put at the forefront of future public psychological provision for HD in Italy.

### 4.3. Strengths, Limitations, and Future Directions

The main strength of our study is its exploration of the subjective experience of psychological support of people affected by HD in Italy, which currently represents a major gap in the literature. The triangulation of the data across both patients and caregivers also offered the opportunity to expand the reach of this investigation and unearth themes which may have been overlooked by focusing solely on people with HD.

However, some limitations should be considered along with the current findings. First, just as including caregivers represented a strength, the lack of clinicians in this study may be considered a limitation. Therefore, future research should aim at triangulating information further by exploring the views of psychologists and other mental health providers working with HD. In addition, an intrinsic limitation of qualitative methods is the reliance on smaller, less representative samples. While such methods were appropriate for the aim of the present study [35], only a small number of premanifest individuals with early-stage HD who were already under the care of the LIRH foundation could be recruited for this investigation. Thus, future qualitative and quantitative research should adopt more representative samples to explore the access and use of public psychological provision by people with HD and their caregivers in Italy.

## 5. Conclusions

Based on the present results, patients and caregivers perceive public psychological support for HD in Italy as unavailable or inadequate, with private therapy being often seen as unaffordable. A number of barriers, including distrust in public healthcare and preconceptions about therapy, may limit access to psychological care. On the other hand, advice from HD organisations, finding the right clinician, improving affordability, and seeking therapy for reasons other than HD appear to act as enablers. Ultimately, future public psychological provision in Italy should aim to provide free or basic fee tailored psychological support to both patients and their family members across all stages of the disease.

## Figures and Tables

**Table 1:** Summary of participants’ characteristics.

Participant ID	Age	Gender	Area of Italy	Time Since Clinical Diagnosis
Patient 1	41	Male	Centre	1 year
Patient 2	61	Female	North	9 years
Patient 3	57	Male	South	1 year
Patient 4	38	Female	Centre	3 years
Patient 5	52	Female	Centre	4 years
Patient 6	51	Female	South	3 years
Patient 7	49	Female	Centre	<1 year
Caregiver 1	42	Female	Centre	
Caregiver 2	68	Male	North	
Caregiver 3	57	Female	South	
Caregiver 4	39	Male	Centre	
Caregiver 5	53	Male	Centre	
Caregiver 6	55	Male	South	
Caregiver 7	52	Male	Centre	

**Table 2:** Summary of the four main topics of the interview schedule.

Topic	Example Question
Introduction and preliminary clinical information	When were you/was your partner diagnosed with Huntington’s disease?
Psychological impact of HD	Have you ever received any information from healthcare professionals on the psychological impact of HD?
Psychological support for HD	Have you ever received any form of public/free psychological support for HD?
Closing remarks	Is there anything you would like to add that we did not have a chance to discuss?

**Table 3:** Summary of the identified themes and relative codes.

Theme	Codes
*‘Unfortunately, that’s our country for you’*: availability of psychological support in HD	Lack of public psychological provision No psychological support at testing Regional disparities in healthcare Support from organisations
*‘I’m afraid of seeing someone who doesn’t know HD’*: barriers to accessing psychological support	Cost of private therapy Distrust in public healthcare Lack of knowledge about HD Preconceptions around therapy Illness denial
*‘I would speak with my HD organisation first’*: enablers to accessing psychological support	Advice from organisations Special or discounted fees Finding the right person Seeking therapy for other reasons
*‘We should just be able to call’*: Future development of public psychological provision in HD	Direct psychological pathways Free or basic fee therapy Tailored psychological provision Psychological support for family members Psychological provision across all stages

## Data Availability

The data supporting the findings of this study are available on request from the corresponding author. The data are not publicly available due to privacy, ethical restrictions, or other concerns.
